# Myeloid-Derived Suppressor Cells as Therapeutic Targets in Uterine Cervical and Endometrial Cancers

**DOI:** 10.3390/cells10051073

**Published:** 2021-04-30

**Authors:** Seiji Mabuchi, Tomoyuki Sasano

**Affiliations:** 1 Department of Obstetrics and Gynecology, Nara Medical University, Nara 634-8522, Japan; 2 Department of Obstetrics and Gynecology, Osaka Saiseikai Nakatsu Hospital, Osaka 530-0012, Japan; sasano106@yahoo.co.jp

**Keywords:** MDSC, ovarian cancer, survival, therapeutic target, tumor microenvironment

## Abstract

Uterine cervical and endometrial cancers are the two most common gynecological malignancies. As demonstrated in other types of solid malignancies, an increased number of circulating or tumor-infiltrating myeloid-derived suppressor cells (MDSCs) have also been observed in uterine cervical and endometrial cancers, and increased MDSCs are associated with an advanced stage, a short survival, or a poor response to chemotherapy or radiotherapy. In murine models of uterine cervical and endometrial cancers, MDSCs have been shown to play important roles in the progression of cancer. In this review, we have introduced the definition of MDSCs and their functions, discussed the roles of MDSCs in uterine cervical and endometrial cancer progression, and reviewed treatment strategies targeting MDSCs, which may exhibit growth-inhibitory effects and enhance the efficacy of existing anticancer treatments.

## 1. Introduction

Uterine cervical and endometrial cancers are the two most common gynecological malignancies. In the United States, 13,800 and 65,620 new cases of cervical and endometrial cancers, respectively, were reported in 2020 [1]. Although surgery followed by tailored adjuvant treatment is potentially curative, a considerable number of patients develop recurrence and die due to disease progression; 4290 and 12,590 deaths due to cervical and endometrial cancers, respectively, were reported in 2020 in the United States [1].

Cervical cancer has been considered an immunogenic tumor, as it is induced by persistent infection with human papillomavirus. Due to the existence of polymerase epsilon–ultramutated and microsatellite instability–hypermutated tumors, endometrial cancer has also been considered immunogenic and a reasonable candidate for active and/or passive immunotherapy [2]. Although immunotherapy (such as that with the programmed death [PD]-1 antibody pembrolizumab) has recently become a viable treatment for cervical and endometrial cancers, it has limited clinical efficacy.

Suppression of tumor immune surveillance is a main mechanism that prevents the destruction of tumor cells by the immune system and limits the efficacy of existing cancer treatments, including radiotherapy, chemotherapy, and immunotherapy [3]. Myeloid-derived suppressor cells (MDSCs) are a heterogeneous population of immature myeloid cells (IMCs) that play a central role in suppressing antitumor immunity. Additionally, MDSCs can directly stimulate tumor cell proliferation, metastasis, and angiogenesis [4]. As all of these can lead to tumor progression during radiotherapy or chemotherapy and limit the potency of current immunotherapy that targets cytotoxic T lymphocyte-associated protein 4 (CTLA-4) or PD-ligand 1 (PD-L1)/PD-1 [5], MDSCs are considered promising therapeutic targets and predictive biomarkers of treatment outcomes in patients with solid malignancies, including gynecological cancers [4,6].

In this review, we have summarized the current knowledge on MDSC biology and its role in uterine cervical and endometrial cancers. In addition, we have discussed the utility of MDSCs as a predictive marker and highlighted the therapeutic targets of MDSCs in patients with uterine cervical and endometrial cancers.

## 2. MDSC Nomenclature

MDSCs were discovered in the late 1970s. At that time, they were regarded as formerly unknown immune cells that possess immunosuppressive features [7]. Owing to their immunosuppressive functions and immature status, they were called immature myeloid, myeloid suppressor, or natural suppressor cells. In 2007, Gabrilovich et al., after 37 years of their discovery, named these cells as ‘‘MDSCs’’ based on their origins and functions [8].

MDSCs are a heterogeneous population of IMCs, and their number is increased in states of cancer, inflammation, or infection. MDSCs differ from terminally differentiated mature myeloid cells (macrophages, dendritic cells (DCs), or neutrophils) and can be subdivided into two major subsets based on their phenotypic and morphological features—monocytic–MDSCs (M–MDSCs) and polymorphonuclear (PMN) MDSCs (PMN–MDSCs, also known as granulocytic MDSCs) [4,8].

In mice, MDSCs are characterized by the expression of glutathione reductase (Gr-1) and CD11b myeloid lineage differentiation markers (CD11b^+^Gr-1^+^ cells). However, as Gr-1 is a combination of lymphocyte antigen (Ly) 6C and Ly6G, M–MDSCs can be further defined as CD11b^+^Ly6C^high^Ly6G^–^ cells, and PMN–MDSCs can be defined as CD11b^+^Ly6C^l^°^w^Ly6G^+^ cells [4,8].

Human MDSCs are positive for CD11b and CD33 and negative for human leukocyte antigen–antigen D related (HLA–DR) and lineage markers (CD3, CD13, CD19, and CD56). PMN–MDSCs express CD15 but not CD14; hence, they are defined as CD11b^+^CD33^+^HLA–DR^−/low^CD14^−^CD15^+^ cells. M–MDSCs express CD14 but not CD15; hence, they are called CD11b^+^CD33^+^HLA–DR^−/low^CD14^+^CD15^−^ cells. In addition to M–MDSCs and PMN–MDSCs, a third small population of MDSCs exhibiting promyelocytic appearance has been described in humans—immature or early-stage MDSCs, defined as CD33^+^CD11b^+^HLA–DR^−^CD14^−^CD15^−^ cells [4,8]. However, recent investigations have suggested that early-stage MDSCs defined by these surface markers include significant number of basophils [9]. Thus, further efforts will be required to define this MDSC subset.

## 3. MDSC Development, Activation, and Recruitment

### 3.1. MDSC Development and Activation

Under normal circumstances, IMCs differentiate into macrophages, neutrophils, and DCs. However, under pathological conditions such as infection, inflammation, or cancer, the differentiation of IMCs is impaired, leading to the formation of MDSCs [4,8]. The development of MDSCs is a complex phenomenon consisting of increased production of IMCs in the bone marrow, inhibition of the terminal differentiation of IMCs, and pathological activation of MDSCs. Multiple factors secreted from cancer or stromal cells, such as macrophage colony-stimulating factor, granulocyte colony-stimulating factor (G-CSF), granulocyte monocyte colony-stimulating factor, vascular endothelial growth factor (VEGF), transforming growth factor-beta (TGF-β), tumor necrosis factor-alpha, prostaglandin E2 (PGE2), interleukins (IL-1β, IL-10, IL-4, and IL-6), and noncoding RNAs (microRNAs and long noncoding RNAs) are involved in these processes [4,8,10]. In addition to these, recent investigations have suggested that tumor-derived exosomes are involved in the development of MDSCs through the communication with bone marrow cells [11].

These factors in MDSCs trigger the activation of the following signaling pathways to stimulate their suppressive activities: signal transducer and activator of transcription 3 (STAT3), nuclear factor–kappa B, phosphoinositide 3-kinase/AKT/mammalian target of rapamycin (PI3K/AKT/mTOR), and CCAAT/enhancer-binding protein β [4,8]. Of these, upregulation of STAT3 and C/EBP-β appears to be the most prominent, as they regulate the expression of arginase and inducible nitric oxide synthase (iNOS) [12]. STAT3 also downregulates interferon-related factor-8, a negative regulator of MDSCs [13].

### 3.2. Recruitment of MDSCs into the Tumor Microenvironment

Chemokines are important factors in the direct migration of MDSCs. Accumulating evidence has demonstrated that multiple chemokines in the tumor microenvironment (TME), including C-X-C motif ligand (CXCL) 1, CXCL8, CXCL12, C-C motif ligand (CCL) 1, CCL2, CCL3, CCL5, CCL7, and their corresponding receptors on MDSCs (C-C chemokine receptor (CCR) 2, CCR5, and C-X-C chemokine receptor 4) differentially regulate the recruitment of MDSCs [4].

### 3.3. Effect of Cancer Treatment on Tumor-Infiltrating MDSC

Radiotherapy and surgery have been curative treatment options in patients with uterine cervical or endometrial cancer. Recent investigations have suggested that radiotherapy has two opposite effects on MDSC recruitment into TME: conventional fractionated radiotherapy increases MDSCs, while ablative hypofractionated radiotherapy decreases MDSCs. In a mouse model of prostate cancer, a fractionated radiotherapy (3 Gy × 5) has been shown to increase MDSC in the tumor, spleen, or lymph nodes via the production of colony stimulating factor 1 [14]. On the other hand, in mice models of colon tumors, a single, high-dose irradiation (30 Gy) has been shown to reduce MDSC infiltration into the TME [15]. This high dose is at the upper end used clinically to treat advanced or metastatic colorectal, liver, and non-small cell lung tumors. Although fractionated radiotherapy has been employed in the treatment, so far, no studies have investigated the effect of radiotherapy on MDSC recruitment in uterine endometrial and cervical cancer.

## 4. Functions of MDSCs

### 4.1. Immunosuppressive Functions of MDSCs

MDSCs suppress T cells in both antigen-specific and antigen-nonspecific ways by utilizing several mechanisms. The most prominent factors include arginase-1 (Arg-1), nitric oxide (NO), and reactive oxygen species (ROS). Of these, ROS is responsible for antigen-specific suppression that requires close contact of MDSCs and T cells, as ROS are unstable and active only for a very short period. In contrast, NO and Arg-1 that have relatively longer half-life, are responsible for antigen-nonspecific suppression.

**Figure 1:**
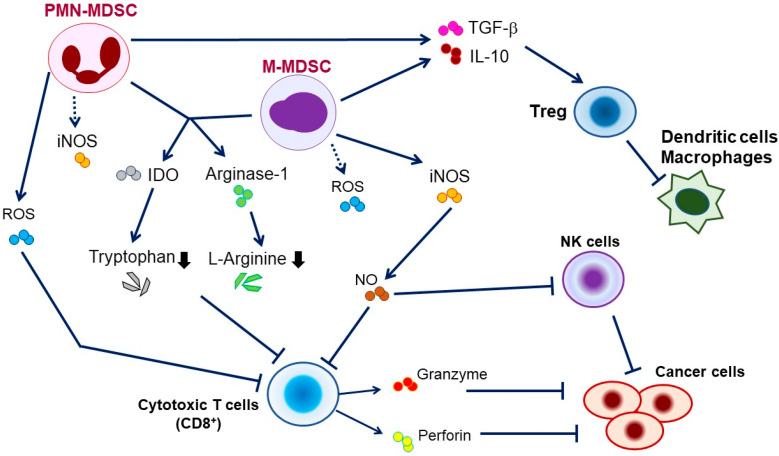
Immune suppression by myeloid-derived suppressor cells (MDSCs) in the tumor microenvironment. Immune suppression by MDSCs is mainly antigen-specific, contact-dependent, and utilizes several major pathways, such as (a) production of reactive oxygen (ROS) and reactive nitrogen species (nitric oxide (NO) or peroxynitrite (PNT)); (b) elimination of L-arginine or L-tryptophan, key nutrition factors for T cells, from the tumor microenvironment by the production of arginase-1 or indoleamine-2,3-dioxygenase (IDO), respectively; (c) disruption of homing of T cells (through the expression of ADAM17); (d) production of immunosuppressive cytokines (transforming growth factor (TGF)-β, interleukin [IL]-10); and (e) induction of T regulatory (Treg) cells.

Both PMN– and M–MDSCs produce Arg-1 (Figure 1), which causes the removal of L-arginine, an essential amino acid for T cell differentiation, from the TME. The depletion of L-arginine subsequently causes the downregulation of CD247 (the ζ-chain of the T cell receptor) expression in T cells. As CD247 is a subunit of the natural killer (NK) receptors NKp46, NKp30, and TcγIII in NK cells, the depletion of L-arginine leads to the inhibition of T cell and NK cell proliferation [4,8].

PMN–MDSCs have increased NADPH oxidase activity and produce large amounts of ROS, which lead to the production of peroxynitrite (PNT). As ROS and PNT are unstable and have very short half-life, PMN–MDSCs require close cell-to-cell contact to exert their effect on T cells. During the close interaction between MDSCs and CD8+ T cells via antigen recognition, PNT causes nitration and conformational changes of the TCR complex. CD8+ T cells consequently lose their binding ability to peptide–MHC class I complex and become nonresponsive to specific peptide presented by tumor cells. PNT may also induce nitration and structural changes of MHC class I molecules on tumor cells, leading to reduced capacity of antigenic peptide binding and impairment of recognition of tumor cells by CD8+ T cells [16,17].

M–MDSCs show low ROS production; however, they express high levels of iNOS, which produces NO that nitrates signaling molecules downstream of FcgRIIIA, resulting in the inhibition of the activities of T cells and NK cells [4,8]. NO also downregulates JAK3/STAT5 signaling, which is crucial for the survival of T cells and NK cells, leading to apoptosis or diminished interferon response [18]. Owing to the fact that NO has a much longer half-life than ROS, it is believed that M–MDSCs have higher suppressive activity than PMN–MDSCs when assessed on a per-cell basis [19].

Other roles of MDSCs in immune suppression include the production of indoleamine-2,3-dioxygenase, which decreases tryptophan levels in the TME, leading to the induction of cell cycle arrest or apoptosis of T cells. MDSCs can also produce immunosuppressive cytokines such as IL-10 and TGF-β, affect NK cell function, and induce regulatory T cell (Treg) expansion [4]. Lastly, MDSCs have an increased expression of PD-L1, which leads to the downregulation of T cell function via engagement of cell surface PD-1 [20].

### 4.2. Nonimmune Functions of MDSCs

In addition to immune-suppressive mechanisms, MDSCs promote cancer progression by stimulating tumor angiogenesis, promoting invasiveness by facilitating epithelial-to-mesenchymal transition or enhancing the metastatic activity of cancer cells by creating “premetastatic niches” [21,22,23]. These processes are regulated by MDSC-derived mediators, including VEGF, basic fibroblast growth factor, Bv8, S100A8/A9, and matrix metalloproteinase-9 [4,21]. Moreover, MDSCs can enhance the stem-like properties of cancer cells, which might mediate resistance to anticancer treatments, including chemotherapy or radiotherapy [24].

## 5. MDSCs in Patients with Solid Cancers

An increased number of circulating MDSCs has been detected in various patients with cancers. In most cancers, including lung, breast, colon, renal, head and neck, and pancreatic cancers, PMN–MDSCs represent the major population of MDSCs. However, patients with melanoma, multiple myeloma, and prostate cancer have a substantially higher proportion of M–MDSCs in the peripheral blood than that of PMN–MDSCs [25].

According to previous reports, an increased number of pretreatment MDSCs has been associated with advanced clinical stage, high probability of recurrence, and short survival [4,6,8,19]. Moreover, a recent investigation has suggested that an increased number of pretreatment MDSCs may be a reliable predictor of poor response to immune checkpoint inhibitors (ICIs), including anti-PD-1, anti-PD-L1, and anti-CTLA-4 agents [26].

## 6. MDSCs in Patients with Uterine Cervical and Endometrial Cancers

### 6.1. Nonpregnant Condition

#### 6.1.1. Findings from Laboratory Investigations

In preclinical investigations (Table 1), cervical cancer cells have been shown to induce MDSCs from PBMCs of healthy donors. Further, a co-culture experiment indicated that MDSCs in patients with cervical cancer can be induced by tumor-derived factors [27]. Factors that induced MDSC in mice models of uterine endometrial and cervical cancer include tumor-derived G-CSF, IL-6, estradiol (E2), and Swainsonine [21,22,28,29,30,31,32,33,34]. MDSCs obtained from these experimental models have been shown to inhibit the activity of CD8^+^ T cells [21,22,28,29,30,31,35], stimulate tumor angiogenesis [21], contribute to premetastatic niche formation [22,23], and increase the stem-like properties of cancer cells [29,30], all of which can promote tumor progression by facilitating tumor growth, metastasis [22], and resistance to anticancer treatments, including chemotherapy and radiotherapy [21,28].

#### 6.1.2. Findings from Patients

In 2014, Mabuchi et al. [21] and Vanderstraeten et al. [36] first demonstrated an increased number of MDSCs in patients with cervical and endometrial cancers, respectively (Table 2). Since then, an increasing number of reports have suggested that the number of M–MDSCs and PMN–MDSCs is significantly increased in peripheral blood mononuclear cells (PBMCs) [21,22,28,35,37], lymph nodes [23,38], and tumors [29,30,31,33,36,39] in patients with uterine cervical and endometrial cancers. The ratio of PMN–MDSCs and M–MDSCs is unknown in cervical cancer; however, a previous study has suggested that G–MDSCs are the dominant subset in endometrial cancer [36].

In studies investigating the cause of increased MDSC production in patients with uterine cervical and endometrial cancers, an increased number of MDSCs was found to be associated with increased serum E2 concentrations [33], serum PGE2 concentrations [29,30], leukocytosis [21,22,29], tumor G-CSF concentrations [21,22,28,29], IL-6 expression [29], or number of cancer stem-like cells in tumors [29,30].

Consistent with the findings of in vitro and in vivo experiments, an increase in the number of circulating or tumor-infiltrating MDSCs was associated with a decrease in the number of tumor-infiltrating CD8^+^ T cells [29,31,35]. An increased number of MDSCs was also associated with unfavorable clinicopathological parameters, including advanced clinical stage [35], visceral or lymph node metastases [22,35,38], deep stromal invasion [35], poor sensitivity to anticancer treatments (radiotherapy or chemotherapy) [21,28,29], high recurrence rate [35], and short survival [21,28,29,31] in patients with uterine cervical and endometrial cancers (Table 2). Moreover, it has been recently demonstrated that MDSC-mediated premetastatic niche formation in the lymph nodes induces 18F-fluorodeoxyglucose (FDG) uptake during FDG-positron emission tomography/computed tomography and causes false-positive detection of nodal metastasis [23].

However, abovementioned studies have limitations that warrant further investigation—small sample size, inconsistent histological subtypes, use of inconsistent MDSC surface markers, and limited clinical information.

### 6.2. Pregnant Condition

During pregnancy, maternal plasma levels of estradiol increase up to 100-fold compared to the nonpregnant status. A previous study demonstrated that the exogenous E2 treatment stimulated the mobilization of MDSC from bone marrow and directly augmented their suppressive activities, leading to the progression of cervical cancer [33]. Consistent with this, a significantly increased number of tumor-infiltrating MDSCs was observed in pregnant women with cervical cancer or in pregnant mice bearing human cervical cancer, which can be attributed to the increased E2 levels during pregnancy [33]. These results indicate that E2 facilitates the progression of female cancers, including cervical cancer, under pregnant condition by inducing MDSC.

## 7. Targeting MDSCs in Uterine Cancer

### 7.1. Rationale for Targeting MDSCs in Cancer Treatment

Accumulating preclinical evidence has shown that MDSC inhibition has therapeutic efficacy against various solid malignancies as a monotherapy or as in combination with existing anticancer treatments [4,6]. Although conventional fractionated radiotherapy increases MDSCs, and ablative hypofractionated radiotherapy decreases MDSCs, inhibition of MDSCs has consistently enhanced the antitumor effect of radiotherapy in preclinical studies, regardless of radiotherapy scheme [40]. Moreover, recent preclinical investigations have suggested that the efficacy of ICIs can be enhanced by MDSC inhibition [26]. As some ICIs have been approved or are being tested in clinical trials in patients with uterine cervical and endometrial cancers, MDSC inhibition can be a promising strategy to extend the benefits of chemotherapy, radiotherapy, or immunotherapy in such patients.

### 7.2. Preclinical Investigation of MDSC-Targeting Therapies in Uterine Cancer

Various MDSC-targeting strategies have been evaluated in murine models of uterine cervical and endometrial cancers (Table 2), such as anti-Gr-1 antibody [22,29,31], anti-IL-6 antibody [32], COX-2 inhibitor [29], STAT3 inhibition [32], and splenectomy [21,28]. They demonstrated significant activity in reducing the number of MDSCs from the TME or inhibiting their suppressive activity against CD8^+^ T cells, which leads to the inhibition of tumor growth or metastasis [21,22,29,33], prolongation of survival [31], attenuation of the growth-promoting effect of E2 [33], or enhancement of the efficacies of existing anticancer treatments, including cisplatin therapy [28] or radiotherapy [21]. In addition to the inhibition of the immunosuppressive activity of MDSCs, depletion of MDSCs has been shown to successfully attenuate premetastatic niche formation and inhibit visceral organ metastasis in uterine cervical and endometrial cancers [22]. Moreover, MDSC depletion has been shown to attenuate the induction of cancer stem-like cells and enhance chemosensitivity in uterine cervical [30] and endometrial cancer [29]. In contrast, MDSC increment in TME using either G-CSF or swainsonine (an alpha-mannosidase inhibitor) has been shown to stimulate the progression of uterine cervical or endometrial cancer [21,22,28,29,33,34]. Collectively, these results strongly indicate the significance of MDSCs as therapeutic targets in this patient population.

### 7.3. Strategies to Therapeutically Target Human MDSCs

In murine studies, anti-Gr-1 antibody has been widely used to eliminate MDSCs from the TME. However, anti-Gr-1 antibodies cannot be used clinically, owing to the absence of a Gr-1 homolog in humans. Currently, although no specific inhibitors of human MDSCs have been developed, various strategies to target MDSCs have been proposed; they have shown promising antitumor effects in preclinical models of solid cancers—(1) depletion of MDSCs, (2) MDSC deactivation, (3) inhibition of MDSC recruitment, and (4) promotion of the differentiation of MDSCs into mature cells (Table 3) [41,42,43,44,45,46,47,48,49,50,51,52,53,54,55,56,57,58,59,60,61,62,63,64,65,66,67,68,69,70,71,72,73,74,75,76].

### 7.4. Predictive Biomarkers for MDSC-Targeting Therapy

In previous investigations including uterine cervical and endometrial cancer patients, an increased number of MDSCs were observed only in those who displayed tumor-related leukocytosis (TRL) [21,22,28]. In addition, recently, a ribonucleic acid sequencing analysis revealed that the MDSC signature in patients with cervical cancer in the Cancer Genome Atlas database is associated with leukocytosis [39]. These findings are partially in line with previous studies showing that uterine cervical or endometrial cancer patients exhibiting TRL, neutrophilia, increased neutrophil-to-lymphocyte ratio, or those with tumor expressing G-CSF are associated with decreased survival rate or resistance to radiotherapy or chemotherapy [21,28,29]. Moreover, an increased number of MDSCs were detected in patients with uterine cervical and endometrial cancers whose tumors overexpressed G-CSF [21,22,28]. Consistent with these findings, MDSC-targeting treatments, such as anti-Gr-1 antibody treatment or splenectomy, had significant antitumor effects in mouse models of G-CSF-expressing, TRL-positive cervical and endometrial cancers that exhibited increased MDSC [21,28,29]. These results strongly indicate that leukocyte count, neutrophil count, neutrophil-to-lymphocyte ratio or tumor G-CSF expression, which can be easily assessed by peripheral blood cell count or immunohistochemistry, can be used as a biomarker to predict the sensitivity of MDSC-targeting treatments.

Moreover, recently, it was found that an increased number of MDSCs was associated with increased bone marrow FDG uptake in patients with uterine cervical cancer [31]. Thus, by evaluating bone marrow FDG uptake, we might be able to identify a group of patients with increased MDSC who are candidates for MDSC-targeting agents. To the best of our knowledge, these are the only studies that have attempted to identify biomarkers for MDSC-targeting therapy.

Theoretically, other tumor-derived substances (including cytokines, chemokines, noncoding RNAs, or exosomes) that stimulate the production of MDSC can also be predictive biomarkers. We hope the clinical utility of such biomarkers be evaluated preclinically and clinically in the future, which would enable physicians to identify patients who might benefit from MDSC-targeting therapy.

### 7.5. Clinical Trials Targeting MDSCs in Patients with Solid Cancers

Although various MDSC-targeting strategies have been proposed in preclinical investigations (Table 3), only a few of them are tested in ongoing clinical trials. These agents include capecitabine, gemcitabine, ibrutinib (Bruton tyrosine kinase inhibitor), IPI-549 (PI3K inhibitor), and SX-682 (CXCR1/2 inhibitor) (Table 3). The activity of MDSC inhibition has also been tested in a setting of combination therapy to establish a strategy to overcome resistance to ICIs. The safety, efficacy, and immunobiological effects of the CXCR4 antagonist BL-8040 (motixafortide) with pembrolizumab have recently been evaluated in a phase IIa study of metastatic pancreatic ductal adenocarcinoma (PDAC) [73]. In the study, BL-8040 increased the number of tumor-infiltrating CD8^+^ effector T cells and decreased the number of MDSCs in PDAC tumors, suggesting that CXCR4 inhibition may enhance the therapeutic efficacy of PD-1 blockade in patients with PDAC and warrants confirmation in subsequent randomized trials.

Activating mutation of PIK3CA and the resulting activation of PI3K is frequently observed in both uterine endometrial and cervical cancer, and, thus, PI3K-inhibition has been regarded as promising treatment [77]. Moreover, as CXCR2 has been shown to be involved in the MDSC recruitment into TME of uterine endometrial and cervical cancer [22,29], we hope that the activity of IPI-549 or SX-682 will be evaluated in this patient population. Positive clinical data on MDSC-targeting therapies are anticipated in the future.

## 8. Conclusions

An increased number of MDSCs is observed in patients with uterine cervical and endometrial cancers. MDSCs play a significant role in disease progression. To inhibit their tumor-promoting effects, the efficacy of MDSC-targeting therapies (either as monotherapies or in combination with existing treatments) against uterine cervical and endometrial cancers is currently being evaluated preclinically. We believe that increasing our understanding of MDSC biology will aid in the development of optimal MDSC-targeting therapies for patients with uterine cervical and endometrial cancers.

## Figures and Tables

**Table 1:** Summary of in vitro/in vivo investigations of MDSC in uterine cervical and endometrial cancer.

Author/Year/Type of Cancer	Findings from In Vitro/In Vivo Studies of Uterine Cervical and Endometrial Cancer
Mabuchi, S., et al. 2014 [21] Cervical cancer	MDSC inhibited the activity of CD8^+^ T cells and stimulated angiogenesis. MDSCs were responsible for the rapidly progressive and radioresistant nature of cervical cancer. The administration of anti-Gr-1-neutralizing antibody or the depletion of MDSCs by splenectomy inhibited tumor growth and enhanced radiosensitivity in cervical cancer.
Sasano, T., et al. 2018 [22] Cervical cancer	MDSC inhibited the activity of CD8^+^ T cells. MDSCs were involved in premetastatic niche formation, which promotes visceral organ metastasis. MDSCs created premetastatic niche by expressing high levels of Cxcl2, S100a8/9, Bv8, and MMP-9, which promotes visceral organ metastasis. MDSCs attracted cervical cancer cells to visceral organ via CXCL2/CXCR2 axis. The depletion of MDSCs by anti-Gr-1 antibody attenuated premetastatic niche formation and effectively inhibited the visceral organ metastasis.
Lechner, M.G., et al. 2011 [27] Cervical cancer	Cervical cancer cells induced MDSC (CD33^+^ HLA–DR^low^Lineage^-^) from healthy donor PBMC in a co-culture experiment. Increased expression of transcription factors HIF1α, STAT3, and C/EBPβ were observed in MDSCs.
Kawano, M., et al. 2015 [28] Cervical cancer	MDSC inhibited the activity of CD8^+^ T cells. G-CSF activated MDSC function via G-CSF receptor–STAT3 signaling pathway. Increased MDSC was involved in the development of chemoresistance. The depletion of MDSC via splenectomy or by anti-Gr-1 antibody sensitized cervical cancer to cisplatin.
Yokoi, E., et al. 2020 [29] Endometrial cancer	MDSC inhibited the activity of CD8^+^ T cells. MDSC enhanced stemness of cancer cells by producing PGE2. G-CSF collaborated with IL-6 in stimulating the activities of MDSCs. MDSC depletion using an anti-Gr-1-neutralizing antibody or inhibition of MDSC activity by celecoxib inhibited tumor growth and enhanced chemosensitivity in endometrial cancer.
Kuroda, H., et al. 2018 [30] Cervical cancer	MDSC inhibited the activity of CD8^+^ T cells. MDSC induced by tumor-derived G-CSF enhanced the stemness of cervical cancer cells by producing PGE2. MDSC depletion using an anti-Gr-1-neutralizing antibody or inhibition of MDSC activity by celecoxib inhibited the induction of cancer stem-like cells and enhanced the efficacy of cisplatin in cervical cancer.
Shimura, K., et al. 2021 [31] Cervical cancer	MDSC inhibited the activity of CD8^+^ T cells. MDSC depletion using an anti-Gr-1-neutralizing antibody prolonged the survival of cervical cancer-bearing mice exhibiting increased MDSC.
Lee, B.R., et al. 2016 [32] Cervical cancer	Increased IL-6 was associated with enhanced tumor growth and increased MDSC generation. Anti-IL-6 receptor monoclonal antibody inhibited tumor growth and MDSC generation. STAT3 inhibitor reduced tumor growth, inhibited MDSC expansion, and relieved T cell suppression.
Kozasa, K., et al. 2019 [33] Cervical cancer	Estradiol (E2) stimulated the mobilization of MDSC from bone marrow and augmented their suppressive activities, leading to the progression of cervical cancers. Co-administration of an anti-Gr-1-neutralizing antibody with E2 prevented the E2-mediated induction of MDSC and attenuated E2-mediated tumor growth in cervical cancer xenografts. Significantly increased MDSC and enhanced tumor growth were observed during pregnancy in mice with cervical cancer.
Silveira, C.R.F., et al. 2019 [34] Cervical cancer	Swainsonine, an alpha-mannosidase inhibitor, promoted cervical cancer progression by inducing MDSC, which inhibited T cell activation.
Liang, Y., et al. 2019 [35] Cervical cancer	Patient-derived MDSC inhibited the activity of CD8^+^ T cells.

MDSC, myeloid-derived suppressor cells; CD, cluster of differentiation; Gr-1, glutathione reductase 1; PGE2, prostaglandin E2; G-CSF, granulocyte-colony stimulating factor; IL, interleukin; CXCL, chemokine (C-X-C motif) ligand; S100a8/9, S100 calcium-binding protein a8/9; MMP-9, matrix metalloproteinase 9; CXCR, chemokine (C-X-C motif) receptor; STAT3, signal transducer and activator of transcription 3; HLA–DR, human leukocyte antigen–antigen D related; HIF, hypoxia inducible factor.

**Table 2:** Summary of studies investigating the role of MDSC in uterine cervical and endometrial cancer patients.

Author/Year	Type of Cancer	Samples Examined Marker of MDSC	Findings from Patient-Derived Samples
Mabuchi, S., et al. 2014 [21]	Healthy donner Cervical cancer	PBMC (FCM) HLA–DR^−^CD11b^+^CD33^+^ cells	Increased circulating MDSC was associated with leukocytosis. Tumor G-CSF expression was significantly associated with increased circulating MDSC and compromised survival of cervical cancer patient treated with radiotherapy.
Sasano, T., et al. 2018 [22]	Cervical cancer	PBMC (FCM) HLA–DR^−^CD11b^+^CD33^+^ cells	MDSC in the peripheral blood of cervical cancer patients was positively associated with the number of leukocytes and tumor G-CSF expression.
Mabuchi, S., et al. 2020 [23]	Cervical cancer	Lymph nodes (IHC) CD33^+^ cells	MDSC-mediated premetastatic niche formation in the lymph node of cervical or endometrial cancer patients misled 18F-FDG-PET/CT for detecting nodal metastasis.
Kawano, M., et al. 2015 [28]	Healthy donner Cervical cancer	PBMC (FCM) HLA–DR^−^CD11b^+^CD33^+^ cells	Tumor G-CSF expression was significantly associated with increased circulating MDSC and compromised survival in patients treated with chemotherapy.
Yokoi, E., et al. 2020 [29]	Endometrial cancer	Tumor (IHC) CD33^+^ cells	The number of tumor-infiltrating MDSC was associated with leukocytosis and increased serum PGE2 concentration. The number of tumor-infiltrating MDSC was associated with decreased CD8^+^ T cells in tumor and increased tumor G-CSF or IL-6 expressions. Increased tumor-infiltrating MDSCs was associated increased stemness of endometrial cancer.
Kuroda, H., et al. 2018 [30]	Cervical cancer	Tumor (IHC) CD33^+^ cells	Number of tumor-infiltrating MDSC was positively correlated with the number of cancer stem like cells and serum PGE2 concentration.
Shimura, K., et al. 2021 [31]	Cervical cancer	Tumor (IHC) CD33^+^ cells	Increased MDSCs were associated with increased bone marrow FDG uptake in cervical cancer patients. Increased bone marrow FDG uptake was indicative of poor prognosis.
Kozasa, K., et al. 2019 [33]	Cervical cancer	Tumor (IHC) CD33^+^ cells	Significantly increased MDSC numbers were observed during pregnancy in cervical cancer patients, which can be attributed to the increased estradiol during pregnancy.
Liang, Y., et al. 2019 [35]	Cervical cancer	PBMC (FCM) G–MDSCs: HLA–DR^−^Lin^−^ CD11b^+^CD33^+^CD14^−^CD15^+^ cells M–MDSCs: HLA–DR^−^Lin^−^ CD11b^+^CD33^+^CD14^+^ cells	Increased circulating G/M–MDSCs were observed in cervical cancer patients. Increased circulating MDSC was associated with advanced stage and decreased tumor-infiltrating CD8^+^ T cells. Frequency of circulating G–MDSCs but not M–MDSCs correlated with unfavorable clinicopathologic parameters, including lymph node metastasis, deep stromal invasion, and tumor recurrence.
Vanderstraeten, A., et al. 2014 [36]	Healthy donner Endometrial cancer	Tumor (FCM) G–MDSCs: HLA–DR^−^Lin- CD11b^+^CD33^+^CD14^−^CD15^+^ cells M–MDSCs: HLA–DR^−^Lin-CD11b^+^CD33^+^CD14^+^ CD15^+^ cells	Increased tumor-infiltrating MDSCs and arginase-1 expression were observed in endometrial cancer. Patients-derived G–MDSC and M–MDSC expressed similar levels of arginase-1. G–MDSC was the dominant subset in endometrial cancer.
van Meir, H., et al. 2016 [37]	Cervical cancer	PBMC (FCM) M–MDSCs: CD3^−^CD19^−^CD1a^−^HLA–DR^−^CD14^+^CD15^−^ cells	Radiotherapy was associated with increased circulating M–MDSCs.
Heeren, A.M., et al. 2018 [38]	Cervical cancer	Lymph nodes (FCM) G–MDSCs: HLA–DR^−^ Lin- CD11b^+^CD33^+^CD15^+^ cells M–MDSCs: HLA–DR^−^ Lin- CD11b^+^CD33^+^CD14^+^ cells	Increased M–MDSC was observed in the metastatic lymph nodes than in nonmetastatic lymph nodes. Increased G–MDSC was observed in the metastatic lymph nodes than in nonmetastatic lymph nodes; however, the difference was not statistically significant.
Kim, K.H., et al. 2020 [39]	Cervical cancer	Tumor (RNA sequencing) MDSC signature	MDSC signature in cervical cancer patients in the TCGA database was associated with leukocytosis.

MDSC, myeloid-derived suppressor cells; IHC, Immunohistochemistry; CD, cluster of differentiation; FDG, fluorodeoxyglucose; PET, positron emission tomography; CT, computed tomography; TCGA, The Cancer Genome Atlas; PGE2, prostaglandin E2; G-CSF, granulocyte-colony stimulating factor; IL-6, interleukin 6; PBMC, peripheral blood mononuclear cells; FCM, flow cytometry; G–MDSC, granulocytic MDSC; M–MDSC, monocytic MDSC; HLA–DR, human leukocyte antigen–antigen D related; Lin, lineage.

**Table 3:** Strategies for MDSC-targeting in various types of cancers.

Strategy	Mechanism of Action	Examples	Ongoing Clinical Trials *
(1) Depletion of MDSC	Chemotherapeutic agents	Gemcitabine [41], 5-FU [42], paclitaxel [43], cisplatin [44], docetaxel [45], capecitabine [46], and lurbinectedin [47]	NCT02669173 (Examine the effect of capecitabine on MDSC) NCT01803152 (Examine the effect of gemcitabine on MDSC)
	Tyrosine kinase inhibitors	Sunitinib [48], sorafenib [49], and ibrutinib [50]	NCT03525925 (Examine the effect of ibrutinib on MDSC)
	IL-6 inhibitors	Anti-IL-6R mAb [51]	NA
	CSF1R antagonists	GW2580 [52] and PLX3397 [53]	NA
	S100A9 inhibitors	Tasquinimod [54]	NA
	Diabetes drugs	Metformin [55]	NA
	Thrombin inhibitor	Dabigatran [56]	NA
(2) MDSC deactivation	B-Raf inhibitor	Vemurafenib [57]	NA
	Bisphosphonates	Zoledronic acid [58]	NA
	PDE-5 inhibitors	Sildenafil, tadalafil, and vardenafil [59]	NA
	STAT3 inhibitors	Stattic [60], CPA7 [61], S3I-201 [62], JSI-124 [63], and AG490 [64]	NA
	mTOR inhibitors	Rapamycin [65]	NA
	PI3K inhibitors	IPI-145 [66] and IPI-549 [67]	NCT02637531 (Examine the effect of IPI-549 on MDSC)
	COX2 inhibitors	Celecoxib [29,30]	NA
	NSAID	Nitroaspirin [68]	NA
	HDAC inhibitor	Entinostat [69]	NA
	IDO inhibitor	Indoximod [70]	NA
(3) Prevention of MDSC recruitment	Chemokine receptor antagonists	AZD5069 (CXCR2) [71], Reparixin (CXCR2) [71], SX-682 (CXCR1/2) [71], AMD3100 (CXCR4) [71], CCX872 (CCR2) [72], BL8040 (CXCR4) [73], and Maraviroc (CCR5) [71]	NCT03161431 (Examine the effect of SX-682 on MDSC)
(4) Promoting the differentiation of MDSC	Vitamin A	ATRA [74]	NA
	Vitamin D	1,25(OH)_2_D_3_ [75]	NA
	Casein kinase inhibitor	Tetrabromocinnamic acid [76]	NA
	Chemotherapeutic agents	Paclitaxel [43] and docetaxel [45]	NA

MDSC, myeloid-derived suppressor cells; 5-FU, fluorouracil; IL-6, interleukin 6; IL-6R, interleukin 6 receptor; NA, not applicable; CSF1R, colony stimulating factor 1 receptor; S100A9, S100 calcium-binding protein A9; PDE-5, phosphodiesterase 5; STAT3, signal transducer and activator of transcription 3; mTOR, mammalian target of rapamycin; PI3K, phosphoinositide 3-kinase; COX2, cyclooxygenase 2; NSAID, nonsteroidal anti-inflammatory drug; HDAC, histone deacetylase; IDO, indoleamine 2,3-dioxygenase; CXCR, chemokine (C-X-C motif) receptor; CCR, chemokine (C-C motif) receptor; ATRA, all-trans retinoic acid. * Available from ClinicalTrials.gov; https://www.clinicaltrials.gov/ (accessed on 27 February 2021).

## Data Availability

Not applicable.
